# Genome-wide copy number aberrations and HER2 and FGFR1 alterations in primary breast cancer by molecular inversion probe microarray

**DOI:** 10.18632/oncotarget.14802

**Published:** 2017-01-24

**Authors:** Hui Chen, Rajesh R. Singh, Xinyan Lu, Lei Huo, Hui Yao, Kenneth Aldape, Ronald Abraham, Shumaila Virani, Meenakshi Mehrotra, Bal Mukund Mishra, Alex Bousamra, Constance Albarracin, Yun Wu, Sinchita Roy-Chowdhuri, Rashmi Kanagal Shamanna, Mark J. Routbort, L. Jeffrey Medeiros, Keyur P. Patel, Russell Broaddus, Aysegul Sahin, Rajyalakshmi Luthra

**Affiliations:** ^1^ Departments of Pathology, The University of Texas MD Anderson Cancer Center, Houston, TX, USA; ^2^ Department of Hematopathology, The University of Texas MD Anderson Cancer Center, Houston, TX, USA; ^3^ Department of Bioinformatics and Computational Biology, The University of Texas MD Anderson Cancer Center, Houston, TX, USA; ^4^ Allegheny Health Network, Pittsburgh, PA, USA; ^5^ Department of Anatomic Pathology, Laboratory Medicine Program, University Health Network, Toronto, Canada

**Keywords:** breast cancer, SNP microarray, molecular inversion probe microarray, chromothripsis, HER2, Pathology Section

## Abstract

Breast cancer remains the second leading cause of cancer-related death in women despite stratification based on standard hormonal receptor (HR) and HER2 testing. Additional prognostic markers are needed to improve breast cancer treatment. Chromothripsis, a catastrophic genome rearrangement, has been described recently in various cancer genomes and affects cancer progression and prognosis. However, little is known about chromothripsis in breast cancer. To identify novel prognostic biomarkers in breast cancer, we used molecular inversion probe (MIP) microarray to explore genome-wide copy number aberrations (CNA) and breast cancer-related gene alterations in DNA extracted from formalin-fixed paraffin-embedded tissue. We examined 42 primary breast cancers with known HR and HER2 status assessed *via* immunohistochemistry and FISH and analyzed MIP microarray results for correlation with standard tests and survival outcomes. Global genome-wide CNA ranged from 0.2% to 65.7%. Chromothripsis-like patterns were observed in 23/38 (61%) cases and were more prevalent in cases with =10% CNA (20/26, 77%) than in cases with <10% CNA (3/12, 25%; *p*<0.01). Most frequently involved chromosomal segment was 17q12-q21, the *HER2* locus. Chromothripsis-like patterns involving 17q12 were observed in 8/19 (42%) of *HER2-*amplified tumors but not in any of the tumors without *HER2* amplification (0/19; *p*<0.01). *HER2* amplification detected by MIP microarray was 95% concordant with conventional testing (39/41). Interestingly, 21% of patients (9/42) had fibroblast growth factor receptor 1 (*FGFR1*)amplification and had a 460% higher risk for mortality than those without *FGFR1* amplification (*p*<0.01). In summary, MIP microarray provided a robust assessment of genomic CNA of breast cancer.

## INTRODUCTION

Breast cancer is the most prevalent malignancy and remains the second leading cause of cancer-related death in women [1]. Breast carcinoma is often associated with high-level, complex, genome-wide copy number aberrations (CNA) [2, 3]. Standard biomarker testing for breast cancer includes assessment of estrogen receptor (ER), progesterone receptor (PR), and human epidermal growth factor receptor 2 (HER2) expression by immunohistochemistry (IHC) and *HER2* amplification by fluorescence *in situ* hybridization (FISH) to determine patient eligibility for hormonal and anti-HER2 therapy [4–6]. Breast cancer patients with hormone receptor-positive (HR+) tumors have better clinical outcomes after receiving endocrine therapy than those with HR-negative (HR-) tumors; however, nearly half of ER-positive (ER+) breast cancers do not respond to or develop resistance to hormonal therapy [7–9]. HER2 protein overexpression and gene amplification are present in approximately 15% of breast cancers, and patients with these tumors have an aggressive clinical course [10, 11]. HER2 overexpression/amplification predicts response to effective therapy targeting HER2 [12], however approximately 30% or more patients do not respond to anti-HER2 therapy [13, 14]. Patients with triple-negative breast cancer have the worst clinical outcome, possibly owing to the lack of specific therapeutic targets. Genome-wide mutational analysis and expression profiles have been extensively explored to search for novel therapeutic targets and to identify patients for whom conventional therapy will not be effective [2]. Gene expression profiling has been reported to help predict clinical outcomes for breast cancer patients [7–9]. Fibroblast growth factor receptor 1 (FGFR1) protein expression recently has been associated with poor prognosis of ER+/HER2-negative (HER2-) and triple-negative breast cancer [15–17]. However, alternative robust and reproducible technologies that provide quantitative assessment of *HER2* and *FGFR1* copy number are needed, and application of assays for these and other genes using genome-wide approaches provide a potentially efficient solution for diagnostic labs going forward.

Recent advances in high-throughput molecular technologies have allowed the identification of common targetable genomic alterations in solid tumors, including breast cancer. However, degradation of DNA derived from formalin-fixed paraffin-embedded (FFPE) tissue and the yield of DNA from solid tumors limit the use of many of these new technologies for cancer genome analysis in clinical practice. In this respect, molecular inversion probe (MIP)-based single nucleotide polymorphism microarray technology can provide high-quality genomic data and genome-wide analysis of CNA in solid tumors when only nanograms of degraded DNA are extracted from FFPE tissue [18–20]. In comparison with FISH assay, the conventional standard for detecting single biomarker status such as *HER2* amplification, MIP microarray can provide accurate and quantitative assessment of copy number gain/amplification and loss for nearly 900 cancer related genes as well as copy neutral loss of heterozygosity (LOH) of genes within chromosomal segments [19, 21]. Additionally, MIP microarray can distinguish polysomy *versus* co-segmental amplification of genes and genomic loci that cannot be distinguished by FISH.

Chromothripsis, recently described in cancer genomes, is a single catastrophic event of massive chromosome shattering and complex genomic rearrangement involving localized regions of a single or a few chromosomes [22–24]. Chromothripsis can be modeled and detected by single-nucleotide polymorphism microarray as a distinct genomic pattern with oscillations among 2 or 3 copy number states with tens to hundreds of breakpoints within a localized region and presence of a copy number state alternating between maintenance of heterozygosity *versus* LOH [25, 26]. Chromothripsis has been reported in at least 2%‒5% of all cancers and in 20%‒40% of bone cancers, prostate cancers, and brain tumors [22, 27, 28] and has been reported to involve amplification of oncogenes and inactivation tumor suppressor genes [23, 25]. In certain tumor types, such as neuroblastoma, chromothripsis has been associated with a poor clinical outcome [23]; however, chromothripsis also can be beneficial [29]. Chromothripsis-like patterns have been suggested in breast cancers *via* spectral karyotyping analysis, array comparative genomic hybridization, and whole-genome sequencing [30–35]. Unlike whole genome sequencing, spectral karyotyping analysis and array comparative genomic hybridization cannot provide the allelic information required to distinguish massive chromosome rearrangements owing to a single catastrophic event (chromothripsis) *versus* those owing to progressive rearrangements. Alternating regions of heterozygosity and LOH are present in chromothripsis but are absent in progressive rearrangements [22]. *HER2* copy number assessment in breast cancer by MIP microarray has recently been reported [20, 36]; however, there is limited information on chromothripsis and CNA in breast cancer. Furthermore, additional prognostic markers and potential molecular targets are needed to improve the treatment of patients with breast cancers. We explored global genome-wide CNA in breast cancer by MIP microarray. We examined chromothripsis-like pattern and its association with *HER2*.

## RESULTS

### Genome-wide copy number aberrations

**Figure 1:**
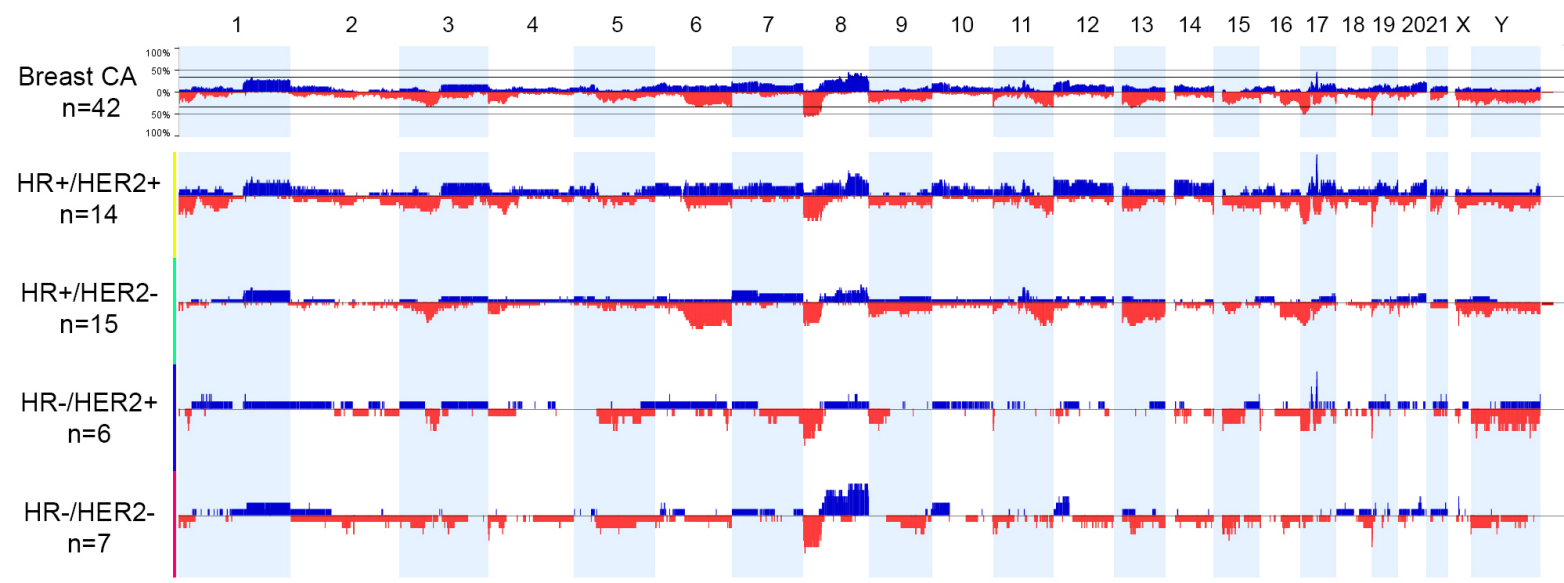
Genome view of copy number status in breast cancer subtypes based on hormonal receptor (HR) and human epidermal growth factor receptor 2 (HER2) status Blue bars indicate the percent of cases with copy number gain (3 or more copies). Red bars indicate the percent of cases with copy number loss. Abbreviations: CA, cancer; HER2+, HER2-positive according to fluorescence *in situ* hybridization; HER2-, HER2-negative and equivocal results according to immunohistochemistry and/or fluorescence *in situ* hybridization; HR-positive according to immunohistochemistry; HR-, HR-negative according to immunohistochemistry.

Genome-wide analysis results showed that CNA (more than 1 copy number gain and any copy number loss) ranged from 0.2% to 65.7% of the genome in all the breast cancer cases examined (mean, 22.7%; median, 18.2%; Figure 1, Table 2). The mean genomic CNA were 29.7% in the HR+/HER2+ group, 19.5% in the HR+/HER2- group, 19% in the HR-/HER2- group, and 16.4% in the HR-/HER2+ group and did not significantly differ among the four subtypes of breast cancer (*p* = 0.15). Genomic analysis revealed substantial copy number gain at 8p11 in the HR+/HER2- group, substantial copy number gain at 8q in the HR+ and HR-/HER2- groups, and substantial copy number gain at 17q12-q21 in the HER2+ group.

**Table 1:** Clinicopathologic features of patients with breast cancer (*N* = 42)

Characteristic	
Age at diagnosis, years	
Median	48
Mean	50
Range	31–74
Gender, n (%)	
Female	41 (98)
Male	1 (2)
Histological type, n (%)	
Ductal	34 (81)
Ductal with micropapillary features	1 (2)
Ductal with mucinous differentiation	1 (2)
Ductal with neuroendocrine differentiation	2 (5)
Ductal with squamous differentiation	1 (2)
Lobular	1 (2)
Mixed ductal and lobular	2 (5)
Tumor nuclear grade, n (%)	
2	9 (21)
3	33 (79)
Nottingham grade, n (%)	
2	10 (24)
3	32 (76)
Primary tumor, n (%)	
T1	21 (50%)
T2	14 (33%)
T3	4 (10%)
T4	3 (7%)
Axillary lymph node metastasise, n (%)	
Absent	21 (50%)
Present	21 (50%)
Distant metastasise, n (%)	
Absent	26 (62%)
Present	16 (38%)
Estrogen receptor ^a^, n (%)	
Positive	29 (69)
Negative	13 (31)
HER2 ^a^, n (%)	
Positive	20 (48)
Equivocal	1 (2)
Negative	21 (50)
Group, n (%)	
HR+/HER2+	14 (33)
HR+/HER2- ^b^	15 (36)
HR-/HER2+	6 (14)
HR-/HER2-	7 (17)
Neoadjuvant therapy, n (%)	14 (33)

^a^Estrogen receptor status was assessed by immunohistochemistry (IHC) analysis; HER2 status was assessed by IHC and/or fluorescence in situ hybridization (FISH) analysis. Both were interpreted according to the American Society of Clinical Oncology/College of American pathologist guidelines with modification [4, 5].

^b^The HR+/HER2- group includes one case with equivocal HER2 results by IHC and FISH.

Abbreviations: HER2, human epidermal growth factor 2; HER2+, HER2‒positive; HER2-, HER2 test with negative and equivocal results; HR, hormone receptor; HR+, HR‒positive; HR-, HR‒negative.

**Table 2:** Genomic change detected by molecular inversion probe microarray in patients with breast cancer (*N* = 38)^a^

	Total	HR+/HER2+	HR+/HER2-	HR-/HER2+	HR-/HER2-
Patients, n (%)	38 (100%)	14 (33%)	12 (36%)	6 (14%)	6 (17%)
Genomic CNA, %					
Range	0.2–65.7	0.2–65.5	3.6–65.7	1–63.9	6.5–38.2
Mean	22.7	29.7	19.5	16.4	19
Median	18.2	25.8	15.9	7.3	16.5
Chromothripsis-like pattern,					
n (%)	23 (61)	10/14 (64)	6/12 (50)	3/6 (50)	4/6 (67)
Genomic CNA<10%	3/12 (25)	0/2 (0)	1/5 (20)	2/4 (50)	0/1 (0)
Genomic CNA≥10%	20/26 (77)^b^	10/12 (83)	5/7 (71)	2/2 (100)	4/5 (80)

^a^Four cases with noisy background due to poor DNA quality were excluded from the analysis for genomic CNA (more than 1 copy number gain and any copy number loss) and chromothripsis-like pattern.

^b^The chromothripsis-like pattern was more prevalent in cases with ≥10% genomic CNA than in cases with <10% genomic CNA (*p* < 0.01).

Abbreviations: CNA, copy number aberrations; HER2, human epidermal growth factor receptor 2; HER2+, HER2–positive according to fluorescence in situ hybridization; HER2-, HER2–negative and equivocal results according to immunohistochemistry and/or fluorescence in situ hybridization; HR, hormone receptor; HR+, HR–positive according to immunohistochemistry; HR-, HR–negative according to immunohistochemistry.

### Chromothripsis-like patterns detected by molecular inversion probe microarray

**Figure 2:**
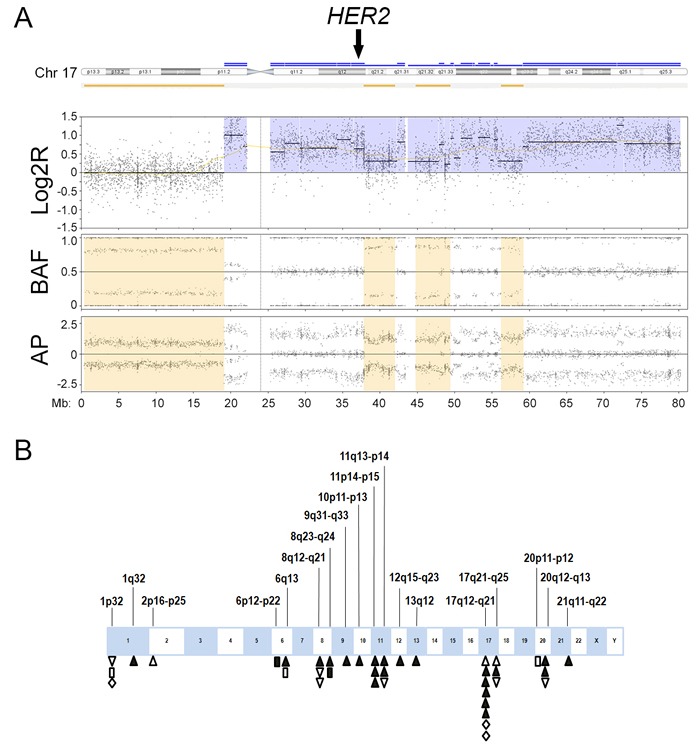
Chromothripsis-like pattern in breast cancer **A**. An example of chromothripsis-like pattern involving the known breast cancer gene human epidermal growth factor receptor 2 (*HER2*) shows the copy number signal in log2R tracing in proximal 17q oscillates between two states (3 to 4 copies), which is confirmed by oscillating changes in the allelic difference pattern in B allelic frequency (BAF) and allelic peak (AP). The oscillation involves more than 10 breakpoints. Chromothripsis-like pattern at 17q12 involves *HER2* amplification (arrow). Log2R, log2 ratio of sample signals to pooled reference. **B**. Distribution of chromothripsis-like pattern in breast cancer. Upright triangles indicate hormone receptor-positive (HR+)/HER2-positive (HER2+) cases; inverted triangles indicate HR+/HER2-negative (HER2-) cases; diamonds indicate HR-negative (HR-)/HER2+ cases; and rectangles indicate HR-/HER2- cases. Open symbols indicate chromothripsis-like pattern involving a single chromosomal segment. Closed symbols indicate chromothripsis-like pattern involving multiple chromosomal segments.

We observed chromothripsis-like patterns involving a single chromosomal segment (*n* = 15) or 2‒5 segments (*n* = 8) in 23 breast cancer cases (61%; Figure 2, Table 2). The most frequently involved segment was 17q12-q21, the locus for *HER2* (*n* = 8), followed by 17q21-q25 (*n* = 3), 8q12-q21 (*n* = 3), 8q23-q24 (*n* = 2), 1p32 (*n* = 3), 11p14-p15 (*n* = 3), 11q13-q14 (*n* = 3), 20q12-q13 (*n* = 3), and 6q13 (*n* = 2). Other single-event loci included 1q23, 2p16-p25, 6p12-p22, 9q31-q33, 10p11-p13, 12q15-q23, 13q12, 20p11-p12, and 21q11-q22. Chromothripsis-like patterns involving multiple chromosomal segments were more frequent in the HR+/HER2+ group (7/10) than in the other groups (1/13; *p* < 0.01). Chromothripsis-like patterns were not detected in the 6q25, 14q23, or 11q22 loci for *ESR1, ESR2*, and *PGR*, respectively. Chromothripsis-like patterns involving any chromosome were more frequent in cases with ≥ 10% genomic CNA (20/26, 77%) than in cases with < 10% CNA (3/12, 25%; *p* < 0.01).

### Concordant interpretation of *HER2* amplification by fluorescence *in situ* hybridization and molecular inversion probe microarray

Of the 20 breast cancer cases positive for *HER2* amplification according to FISH results, 18 (90%) were positive for *HER2* amplification by MIP microarray analysis, using a threshold of 4 and more copies for tumor cells (Table 3). One of the discordant results was due to intratumoral genetic heterogeneity. The second discordant result was a borderline case with an equivocal IHC result (2+) and a borderline positive interpretation for the FISH result (*HER2*/CEP17 ratio, 2.12; average *HER2* copy/cell, 4.65). The only *HER2* case with equivocal IHC and FISH results was not included in the concordance study but was positive for *HER2* amplification by MIP microarray. All 21 breast cancer cases negative for HER2 overexpression by IHC and/or *HER2* amplification by FISH were negative for *HER2* amplification by MIP microarray. MIP microarray's detection of *HER2* amplification status was 95% (39/41) concordant with conventional testing (FISH and IHC) in breast cancer.

**Table 3:** Genetic aberration of breast cancer–related oncogenes and regions detected by MIP microarray

	Total	HR+/HER2+	HR+/HER2-	HR-/HER2+	HR-/HER2-	*P* value
	*n* (%)	*n* (%)	*n* (%)	*n* (%)	*n* (%)	
Patients	42 (100)	14 (33)	15 (36)	6 (14)	7 (17)	
*HER2*						
Amplification by MIP	19 (45)	13 (93)^a^	1 (7)^b^	5 (83)^c^	0	<0.001
CLP at 17q12	8 (19)	6 (43)	0	2 (33)	0	<0.01
17p11.2-q11.2						
Amplification by MIP	14 (33)	8 (57)	1 (7)^b^	5 (83)^c^	0	<0.001
17p11.2 amplification by MIP	5 (12)	4 (29)	0	1 (17)	0	0.06
17q11.2 amplification by MIP	13 (31)	7 (50)	1 (7)^b^	5 (83)	0	<0.001
Co-segmental amplification of 17q12 and 17p11.2-q11.2 by MIP	14 (33)	8 (57)	1 (7)^b^	5 (83)	0	<0.001
*FGFR1*						
Amplification by MIP	9 (21)	3 (21)	3 (20)	1 (17)	2 (29)	1.0

^a^One case showed *HER2* genetic heterogeneity. The immunohistochemistry results were equivocal; fluorescence in situ hybridization results were positive in the tumor area with *HER2–*amplified tumor cells; the overall *HER2* gene status in tumors was negative by MIP microarray.

^b^One case was equivocal for both HER2 overexpression (2+) and *HER2* amplification by fluorescence in situ hybridization (*HER2* copy number/cell, 4.22; *HER2*/CEP17 ratio, 1.1). MIP microarray results were positive for *HER2* amplification (4 copies) and 17q11.2 amplification.

^d^One case was equivocal for HER2 overexpression (2+) and positive for *HER2* amplification by fluorescence in situ hybridization (*HER2* copy number/cell, 4.65; *HER2*/CEP17 ratio, 2.12). MIP microarray results were negative for *HER2* amplification (2.33 copies) and negative for 17p11.2-q11.2 amplification.

Abbreviations: CLP, chromothripsis-like pattern; *FGFR1*, fibroblast growth factor receptor 1; HER2, human epidermal growth factor receptor 2; HER2+, HER2–positive according to fluorescence in situ hybridization; HER2-, HER2–negative and equivocal results according to immunohistochemistry and/or fluorescence in situ hybridization; HR, hormone receptor; HR+, HR–positive according to immunohistochemistry; HR-, HR–negative according to immunohistochemistry; MIP, molecular inversion probe.

### Association of *HER2* amplification with a chromothripsis-like pattern at 17q12

Of the 19 breast cancer cases with *HER2* amplification by MIP microarray, 14 cases (74%) showed co-segmental amplification of both chromosome 17q12, the locus for *HER2*, and 17p11.2-q11.2, the pericentromeric region of chromosome 17 (Table 3). The other five cases (26%) showed segmental amplification of *HER2* without amplification of 17p11.2-q11.2. Of the 23 breast cancer cases negative for *HER2* amplification according to MIP microarray, none showed amplification of 17p11.2-q11.2. Polysomy 17 was not detected in any of the cases. A chromothripsis-like pattern involving 17q12 was observed in eight (42%) HER2+ cases by MIP microarray but was not detected in any of the HER2- cases by MIP microarray (*p* = 0.003; Figure 2, Table 4). Of the 8 cases with chromothripsis-like pattern involving 17q12, five cases showed co-segmental amplification of both *HER2* and 17p11.2-q11.2; the other three cases showed amplification of *HER2* without amplification of 17p11.2-q11.2.

**Table 4:** Correlation of *HER2* amplification and chromothripsis-like pattern at chromosome 17q12 in breast cancer.a

MIP microarray results	*HER2* amplified by MIP (*n*= 19)	*HER2* not amplified by MIP (*n*= 19)
Chromothripsis-like pattern at 17q12 (*n* = 8)	8	0
No chromothripsis-like pattern at 17q12 (*n* = 30)	11	19

^a^*P*=0.003 (Fisher exact test). Four cases with noisy background were excluded from the analysis for chromothripsis-like pattern.

Abbreviations: *HER2*, human epidermal growth factor receptor 2; MIP, molecular inversion probe.

### Correlation of *FGFR1* gene amplification with poor overall survival

**Figure 3:**
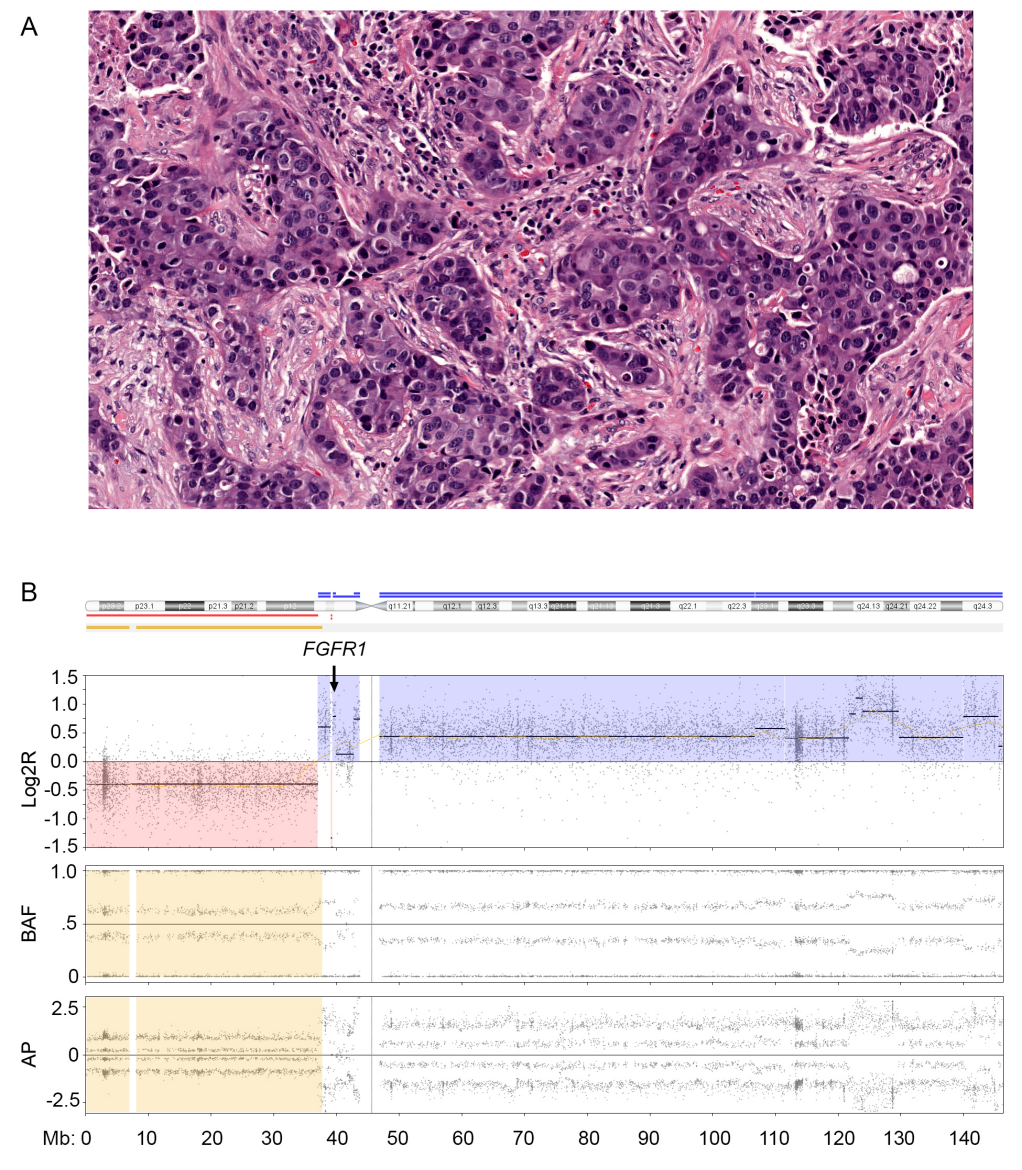
Fibroblast growth factor receptor 1 (FGFR1) amplification in breast cancer **A**. High-grade hormone receptor-negative (HR-)/human epidermal growth factor receptor 2-positive (HER2+) breast cancer (hematoxylin eosin stain; magnification, 20×. **B**. Molecular inversion probe (MIP) microarray analysis of chromosome 8 showed *FGFR1* amplification. The estimated tumor fraction was 40% per MIP microarray analysis similar to that obtained *via* visual estimation by pathologist. Log2R, the log2 ratio; BAF, B allele frequency and AP, allelic peak.

**Figure 4:**
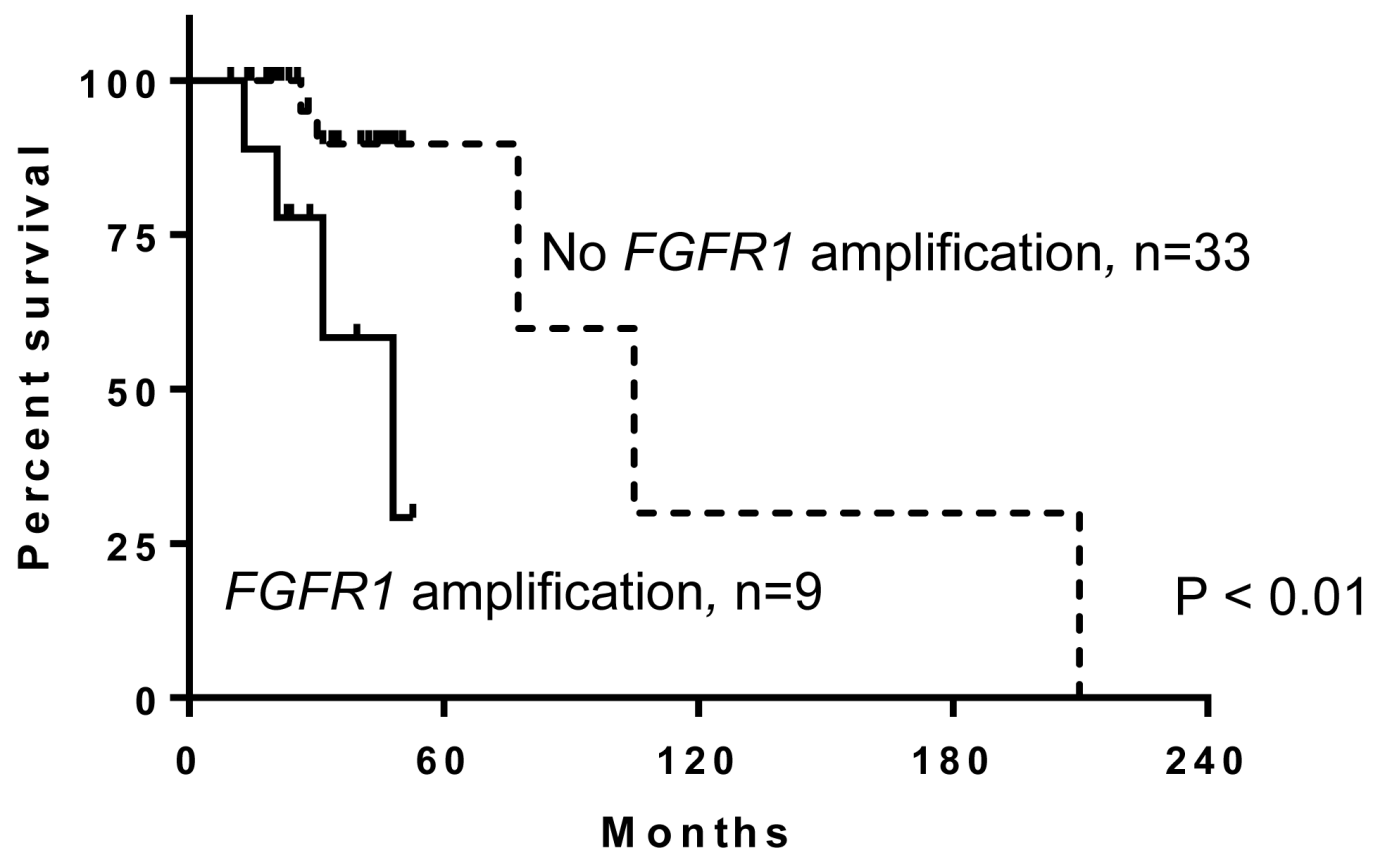
Patients with breast cancer and fibroblast growth factor receptor 1 (*FGFR1*) amplification have worse overall survival than those without *FGFR1* amplification (*p* < 0.01)

Since substantial copy number gain at 8p11 was observed in this study, we assessed for potentially targetable oncogenes in 8p11 and observed *FGFR1* amplification by MIP microarray in nine of 42 cases (21%). Seventeen of the 42 cases including 5 that show *FGFR1* amplification by MIP were also analyzed by NGS as part of clinical testing (Figure 3). NGS confirmed high level of *FGFR1* amplification detected by MIP microarray ( > 8 copies) in 4 cases; however failed to detect low level of *FGFR1* amplification detected by MIP microarray (4 copies) in one case (Supplementary Table 1). *FGFR1* amplification was observed in all four subtypes of breast cancer (Table 3). Breast cancer patients with *FGFR1* amplification had a 460% higher risk of mortality (95% confidence interval, 2.172-87.28; log-rank test) than those without *FGFR1* amplification. Breast cancer patients with *FGFR1* amplification also had worse overall survival (median survival, 48.1 months) than patients without *FGFR1* amplification (median survival, 104.9 months; *p* < 0.01; Figure 4).

## DISCUSSION

In this study, we investigated for genomic CNA in breast cancer using MIP microarray. Consistent with the results of a previous report [20], we found that MIP microarray results provided accurate and quantitative assessment of HER2 status and were 95% concordant with conventional FISH and IHC results. We also observed consistent results between MIP microarray and NGS in detecting *HER2* and *FGFR1* amplification in breast cancer, which supports findings from a previous study [36].

Compared with FISH, MIP microarray minimizes operator counting bias and distinguishes polysomy 17 from co-amplification of *HER2* and the pericentromeric region of chromosome 17. Operator counting bias not infrequently encountered in FISH assay may contribute to the discrepant HER2 interpretations in clinical practice and ultimately affect treatment decision. MIP microarray assay is automated for signal detection and thus avoids counting bias introduced from operators in FISH assay. FISH analysis frequently misinterprets increased counts from both *HER2* and CEP17 probes as a polysomy-like pattern. Several reports have attempted to use FISH analysis to distinguish co-amplification of *HER2* and the pericentromeric region of chromosome 17 from true polysomy 17 using multiple probes on chromosome 17 (tumor protein p53 [*TP53*], topoisomerase II alpha [*TOP2A*], and retinoic acid receptor alpha [*RARA*]) and probes on other chromosomes [37, 38]. Some probes such as *TOP2A* in 17q21-q22, near the *HER2* locus at 17q12, may provide information about molecular targets for anticancer therapy; however, confirming aneusomy 17 *via* multiple FISH probes is challenging without using comparative genomic hybridization or single-nucleotide polymorphism microarray. Indeed, we observed co-segmental amplification of *HER2* and the pericentromeric 17p and/or 17q region in 74% of *HER2*‒amplified cases according to MIP microarray; none of these cases were true polysomy 17, which supports previous studies’ conclusions that true polysomy 17 in breast cancer is rare [37, 39, 40]. Next-generation sequencing (NGS) is a powerful platform for multiplex mutational analysis and for the detection of high-level amplification ( ≥ 8 copies) and deletion. However, the lower level amplification (6-7 copies) detected by FISH may not be detected by NGS if the tumor fraction is low (20%-30%) [41]. MIP microarray, a multiplex platform, shows promise in detecting both high-level and low-level amplification detected by FISH.

As MIP microarray integrates the whole genome, it can provide information on chromothripsis-like pattern. There are few reports about chromothripsis in breast cancer [31, 32, 34] and little information about the effect of chromothripsis on clinical outcomes. In this study, chromothripsis-like patterns occurred in 61% of breast cancer cases in all four subtypes and most frequently involved the 17q12 locus for *HER2,* which was associated with amplification of *HER2*. Some cases with co-amplification of *HER2* and the pericentromeric region of chromosome 17 also had a chromothripsis-like pattern. Owing to the limited sample size and short follow-up, survival analysis did not show survival benefit of HER2+ cases with a chromothripsis-like pattern. The clinical significance of this finding needs to be further evaluated in a large-scale study to assess the ability of genomic profiling to provide prognostic and therapeutic information beyond conventional biomarker analysis.

Notably, we found that amplification of *FGFR1* was associated with increased risk of mortality and poor overall survival. Owing to the limited sample size and events, multivariate analysis could not be performed. Previous studies have reported FGFR1 overexpression and amplification in breast cancer [42–44]. A few reports showed that patients with FGFR1 overexpression in HR+/HER2- and triple-negative breast cancers had poor outcomes [15–17]. Association of *FGFR1* amplification status and clinical outcome has not been reported. Identifying *FGFR1* status could provide prognostic information and could help determine eligibility for targeted therapy, especially for patients with HR+/HER2- and triple-negative breast cancer.

Although MIP microarray shows promise in using small amount of FFPE tissue or fine needle aspirate tissue to detect both high-level and low-level genome-wide amplification without operator bias, MIP microarray is not designed to identify intratumoral heterogeneity and is not able to report the copy number for subpopulations of tumor cells with an amplified oncogene. MIP microarray can only report the averaged signals from tumor cells with both amplified and non-amplified oncogenes when intratumoral heterogeneity is present. In contrast, intratumoral heterogeneity can be identified by careful observers using FISH technology as individual tumor cells are counted for the final score. This may explain the discrepancy we observed in one case with intratumoral heterogeneity with 10% tumor cells staining for HER2 by IHC; *HER2* amplification was interpreted as negative by MIP but positive by FISH.

In conclusion, genomic analysis using MIP microarray provides accurate and quantitative assessment of breast cancer-related oncogenes including *HER2* and *FGFR1*. Global genomic CNA and chromothripsis-like patterns occur frequently in advanced breast cancer. Amplification of *HER2* is associated with a chromothripsis-like pattern at 17q12. *FGFR1* amplification is associated poor clinical outcomes. The global assessment of genomic CNA and chromothripsis-like patterns in a clinical molecular diagnostic laboratory may provide comprehensive information on prognosis and potential benefit from targeted therapy.

## MATERIALS AND METHODS

### Characteristics of patients and samples

We included 42 patients with primary invasive breast carcinoma of intermediate or high nuclear grade and Nottingham histological grade 2 or 3 who underwent biopsy (*n* = 6) or surgery (*n* = 36) between May 2005 and September 2014 and whose tumors were included in the validation studies for breast cancer *HER2* copy number and solid tumor copy number assessment [20, 36]. Table 1 summarizes patients’ demographic, clinical, and pathological information [45]. On the basis of HR expression by IHC and HER2 overexpression and amplification determined by IHC and FISH, we classified the cases into four groups: HR+/HER2+ (*n* = 14), HR+/HER2- (*n* = 15), HR-/HER2+ (*n* = 6), and HR-/HER2- (*n* = 7). HR+ was defined as ≥ 10% tumor cells positive for either ER or PR modified from American Society of Clinical Oncology (ASCO)/College of American Pathologists (CAP) guidelines [4] since patients with low level of ER or PR overexpression (1-9%) were treated similarly to patients with negative results. HER2 overexpression and amplification was interpreted according to the ASCO/CAP guidelines [5]. The two HER2+ groups included 20 HER2+ cases according to FISH results; the remaining 21 HER2- cases according to IHC or FISH and one case with equivocal IHC and FISH results were included in the HER2- groups. Clinical follow-up ranged from 10 months to 18 years from the time of diagnosis (mean, 38 months; median, 31 months). This study was approved by the Institutional Review Board of The University of Texas MD Anderson Cancer Center.

### Immunohistochemistry analysis

We obtained the ER, PR, and HER2 expression status from the medical records. Monoclonal antibodies ER clone 6F11 (Leica Biosystems, Buffalo Grove, IL), PR clone PgR 1294 (Dako, Carpinteria, CA), and HER2 clone e2-4001 (Thermo Fisher Scientific, Waltham, MA) were used to detect the alpha forms of ER, PR, and HER2, respectively.

### Gene amplification detection assays

We obtained FISH based *HER2* amplification status from the medical records and the previous validation study [20]. FISH analysis was performed with a dual-color PathVysion HER-2 DNA Probe kit (Abbott Molecular, Des Plaines, IL) using standard laboratory procedures according to the manufacturer's recommendations. Additionally, we also reviewed amplification status of *HER2* and *FGFR1* generated by NGS as part of routine clinical testing. NGS was performed using an Ion Torrent Personal Genome Machine and AmpliSeq Cancer Hotspot panel v2 (50 genes; Thermo Fisher Scientific). Linear copy number was assessed and based on the normalized coverage depth for the gene of interest in the sample compared with that in the population in our lab‒developed OncoSeek database [46, 47].

### Molecular inversion probe microarray analysis

We circled invasive carcinoma on hematoxylin and eosin-stained slides as a visual reference and manually micro-dissected tumor tissue from consecutive unstained sections of FFPE blocks for genomic DNA extraction and purification using PicoPure DNA extraction kit (ThermoFisher Scientific) and Agencourt AMPureXP kit (Beckman Coulter, Brea, CA) [19]. DNA was quantified using Qubit DNA HS assay Kit (ThermoFisher Scientific). We subjected genomic DNA (50‒80 ng) to MIP microarray using an OncoScan FFPE Assay kit and processed the genome data with OncoScan Console software, version 1.1 (Affymetrix, Santa Clara, CA) [18, 20, 48].

OncoScan Console software reports the actual value of copy number for either tumor cells only or mixed tumor and normal stromal cells. The true copy number for only tumor cells is reported when the percent of aberrant cells is computed. The average copy number of tumor and normal stromal cells within a selected area is reported when the tumor genome is either homogeneous and not distinct from normal stromal cells or too heterogeneous to compute tumor percentage accurately; thus, the percent of aberrant cells is reported either as homogeneous or as not available (NA), respectively. Data visualization of genomic copy number and LOH was performed by Chromosome Analysis Suite (Affymetrix) and Nexus Copy Number software, version 7.5 (BioDiscovery, El Segundo, CA).

### Molecular inversion probe microarray data analysis

We used Nexus Copy Number software for individual case and group analysis to detect genome-wide CNA and chromothripsis-like pattern. The percentage of genomic change from the normal baseline (2 copies) was recorded to include copy number gains greater than 1 copy and any copy number losses. Copy number gains less than 1 copy were excluded from the percentage of genome change to minimize baseline noise. Gene amplification was defined with a designated cut-off of 4 copies for tumor cells [20, 49]. Copy number loss was recorded as 1 copy for tumor cells. Polysomy was recorded when the copy number gain involved the entire chromosome. LOH was recorded if there was hemizygous loss (copy number of 1) for tumor cells or copy neutral LOH in tumor cells with a copy number of 2. When the average copy number of tumor and normal stromal cells (CN_AVE_) was reported, the true copy number for tumor cells (CN_T_) was approximated on the basis of the visual estimate of tumor percentage by the pathologist (%T) using the following formula: CN_T_ = (CN_AVE_ - (1 - %T) × 2) / %T. However, it should be noted that this formula cannot address the issue associated with intratumoral heterogeneity.

Chromothripsis-like patterns were recorded using modified criteria from previous reports [22, 24]: (1) copy number oscillations of 2 or 3 copy number states deviated significantly from normal baseline variation; (2) copy number oscillation involved 10 or more potential breaks; (3) localized copy number oscillation was within 20 Mb of a chromosomal segment; and (4) copy number states alternated between heterozygosity and LOH.

### Statistical analysis

We correlated gene copy number and chromothripsis-like pattern status with gene expression level, gene amplification by conventional assays, and various clinical parameters. Bivariate analysis between categorical variables was performed using the Fisher exact test. Survival analysis was performed using the log-rank test using GraphPad Prism 6 software (GraphPad Software, La Jolla, CA). The cutoff value for statistical significance was 0.05.

## SUPPLEMENTARY MATERIALS TABLE



## References

[1] Siegel RL, Miller KD, Jemal A (2016). Cancer statistics, 2016. CA Cancer J Clin.

[2] Cancer Genome Atlas N (2012). Comprehensive molecular portraits of human breast tumours. Nature.

[3] Desmedt C, Zoppoli G, Gundem G, Pruneri G, Larsimont D, Fornili M, Fumagalli D, Brown D, Rothe F, Vincent D, Kheddoumi N, Rouas G, Majjaj S (2016). Genomic Characterization of Primary Invasive Lobular Breast Cancer. Journal of clinical oncology.

[4] Hammond ME, Hayes DF, Dowsett M, Allred DC, Hagerty KL, Badve S, Fitzgibbons PL, Francis G, Goldstein NS, Hayes M, Hicks DG, Lester S, Love R (2010). American Society of Clinical Oncology/College Of American Pathologists guideline recommendations for immunohistochemical testing of estrogen and progesterone receptors in breast cancer. Journal of clinical oncology.

[5] Wolff AC, Hammond ME, Hicks DG, Dowsett M, McShane LM, Allison KH, Allred DC, Bartlett JM, Bilous M, Fitzgibbons P, Hanna W, Jenkins RB, Mangu PB (2013). Recommendations for human epidermal growth factor receptor 2 testing in breast cancer: American Society of Clinical Oncology/College of American Pathologists clinical practice guideline update. Journal of clinical oncology.

[6] Bardou VJ, Arpino G, Elledge RM, Osborne CK, Clark GM (2003). Progesterone receptor status significantly improves outcome prediction over estrogen receptor status alone for adjuvant endocrine therapy in two large breast cancer databases. Journal of clinical oncology.

[7] Li S, Shen D, Shao J, Crowder R, Liu W, Prat A, He X, Liu S, Hoog J, Lu C, Ding L, Griffith OL, Miller C (2013). Endocrine-therapy-resistant ESR1 variants revealed by genomic characterization of breast-cancer-derived xenografts. Cell reports.

[8] Paik S, Shak S, Tang G, Kim C, Baker J, Cronin M, Baehner FL, Walker MG, Watson D, Park T, Hiller W, Fisher ER, Wickerham DL (2004). A multigene assay to predict recurrence of tamoxifen-treated, node-negative breast cancer. The New England journal of medicine.

[9] LJ van ‘t Veer, Dai H, van de Vijver MJ, He YD, Hart AA, Mao M, Peterse HL, van der Kooy K, Marton MJ, Witteveen AT, Schreiber GJ, Kerkhoven RM, Roberts C (2002). Gene expression profiling predicts clinical outcome of breast cancer. Nature.

[10] Slamon DJ, Clark GM, Wong SG, Levin WJ, Ullrich A, McGuire WL (1987). Human breast cancer: correlation of relapse and survival with amplification of the HER-2/neu oncogene. Science.

[11] Dressler LG, Berry DA, Broadwater G, Cowan D, Cox K, Griffin S, Miller A, Tse J, Novotny D, Persons DL, Barcos M, Henderson IC, Liu ET (2005). Comparison of HER2 status by fluorescence in situ hybridization and immunohistochemistry to predict benefit from dose escalation of adjuvant doxorubicin-based therapy in node-positive breast cancer patients. Journal of clinical oncology.

[12] Cobleigh MA, Vogel CL, Tripathy D, Robert NJ, Scholl S, Fehrenbacher L, Wolter JM, Paton V, Shak S, Lieberman G, Slamon DJ (1999). Multinational study of the efficacy and safety of humanized anti-HER2 monoclonal antibody in women who have HER2-overexpressing metastatic breast cancer that has progressed after chemotherapy for metastatic disease. Journal of clinical oncology.

[13] Perez EA, Romond EH, Suman VJ, Jeong JH, Sledge G, Geyer CE, Martino S, Rastogi P, Gralow J, Swain SM, Winer EP, Colon-Otero G, Davidson NE (2014). Trastuzumab plus adjuvant chemotherapy for human epidermal growth factor receptor 2-positive breast cancer: planned joint analysis of overall survival from NSABP B-31 and NCCTG N9831. Journal of clinical oncology.

[14] Gianni L, Eiermann W, Semiglazov V, Lluch A, Tjulandin S, Zambetti M, Moliterni A, Vazquez F, Byakhov MJ, Lichinitser M, Climent MA, Ciruelos E, Ojeda B (2014). Neoadjuvant and adjuvant trastuzumab in patients with HER2-positive locally advanced breast cancer (NOAH): follow-up of a randomised controlled superiority trial with a parallel HER2-negative cohort. The Lancet Oncology.

[15] Tomiguchi M, Yamamoto Y, Yamamoto-Ibusuki M, Goto-Yamaguchi L, Fujiki Y, Fujiwara S, Sueta A, Hayashi M, Takeshita T, Inao T, Iwase H (2016). FGFR1 protein expression is associated with prognosis in ER-positive/HER2-negative primary breast cancer. Cancer science.

[16] Cheng CL, Thike AA, Tan SY, Chua PJ, Bay BH, Tan PH (2015). Expression of FGFR1 is an independent prognostic factor in triple-negative breast cancer. Breast cancer research and treatment.

[17] Shi YJ, Tsang JY, Ni YB, Chan SK, Chan KF, Tse GM (2016). FGFR1 is an adverse outcome indicator for luminal A breast cancers. Oncotarget.

[18] Wang Y, Carlton VE, Karlin-Neumann G, Sapolsky R, Zhang L, Moorhead M, Wang ZC, Richardson AL, Warren R, Walther A, Bondy M, Sahin A, Krahe R (2009). High quality copy number and genotype data from FFPE samples using Molecular Inversion Probe (MIP) microarrays. BMC medical genomics.

[19] Singh RR, Mehrotra M, Chen H, Almohammedsalim AA, Sahin A, Bosamra A, Patel KP, Routbort MJ, Lu X, Ronald A, Mishra BM, Virani S, Medeiros LJ (2016). Comprehensive Screening of Gene Copy Number Aberrations in Formalin-Fixed, Paraffin-Embedded Solid Tumors Using Molecular Inversion Probe-Based Single-Nucleotide Polymorphism Array. The Journal of molecular diagnostics.

[20] Bousamra AC, Luthra H, Lu R, Aldape X. Y., Singh K, Lu R, Abraham G, Virani R, Mishra S, Sahin B. M., A (2015). Molecular Inversion Probe (MIP) Technology Generates High-Quality HER2 Copy Number Data in Formalin-Fixed Paraffin-Embedded (FFPE) Breast Cancer Tissue. Journal of Cancer and Clinical Oncology.

[21] Foster JM, Oumie A, Togneri FS, Vasques FR, Hau D, Taylor M, Tinkler-Hundal E, Southward K, Medlow P, McGreeghan-Crosby K, Halfpenny I, McMullan DJ, Quirke P (2015). Cross-laboratory validation of the OncoScan(R) FFPE Assay, a multiplex tool for whole genome tumour profiling. BMC medical genomics.

[22] Stephens PJ, Greenman CD, Fu B, Yang F, Bignell GR, Mudie LJ, Pleasance ED, Lau KW, Beare D, Stebbings LA, McLaren S, Lin ML, McBride DJ (2011). Massive genomic rearrangement acquired in a single catastrophic event during cancer development. Cell.

[23] Molenaar JJ, Koster J, Zwijnenburg DA, van Sluis P, Valentijn LJ, van der Ploeg I, Hamdi M, van Nes J, Westerman BA, van Arkel J, Ebus ME, Haneveld F, Lakeman A (2012). Sequencing of neuroblastoma identifies chromothripsis and defects in neuritogenesis genes. Nature.

[24] Korbel JO, Campbell PJ (2013). Criteria for inference of chromothripsis in cancer genomes. Cell.

[25] Leibowitz ML, Zhang CZ, Pellman D (2015). Chromothripsis: A New Mechanism for Rapid Karyotype Evolution. Annual review of genetics.

[26] Stephens PJ, McBride DJ, Lin ML, Varela I, Pleasance ED, Simpson JT, Stebbings LA, Leroy C, Edkins S, Mudie LJ, Greenman CD, Jia M, Latimer C (2009). Complex landscapes of somatic rearrangement in human breast cancer genomes. Nature.

[27] Kovtun IV, Murphy SJ, Johnson SH, Cheville JC, Vasmatzis G (2015). Chromosomal catastrophe is a frequent event in clinically insignificant prostate cancer. Oncotarget.

[28] Kloosterman WP, Koster J, Molenaar JJ (2014). Prevalence and clinical implications of chromothripsis in cancer genomes. Current opinion in oncology.

[29] McDermott DH, Gao JL, Liu Q, Siwicki M, Martens C, Jacobs P, Velez D, Yim E, Bryke CR, Hsu N, Dai Z, Marquesen MM, Stregevsky E (2015). Chromothriptic cure of WHIM syndrome. Cell.

[30] Kim TM, Xi R, Luquette LJ, Park RW, Johnson MD, Park PJ (2013). Functional genomic analysis of chromosomal aberrations in a compendium of 8000 cancer genomes. Genome research.

[31] Xiang DB, Wei B, Abraham SC, Huo L, Albarracin CT, Zhang H, Babiera G, Caudle AS, Akay CL, Rao P, Zhao YJ, Lu X, Wu Y (2014). Molecular cytogenetic characterization of mammary neuroendocrine carcinoma. Human pathology.

[32] Przybytkowski E, Lenkiewicz E, Barrett MT, Klein K, Nabavi S, Greenwood CM, Basik M (2014). Chromosome-breakage genomic instability and chromothripsis in breast cancer. BMC genomics.

[33] Hicks J, Krasnitz A, Lakshmi B, Navin NE, Riggs M, Leibu E, Esposito D, Alexander J, Troge J, Grubor V, Yoon S, Wigler M, Ye K (2006). Novel patterns of genome rearrangement and their association with survival in breast cancer. Genome research.

[34] Tang MH, Dahlgren M, Brueffer C, Tjitrowirjo T, Winter C, Chen Y, Olsson E, Wang K, Torngren T, Sjostrom M, Grabau D, Bendahl PO, Ryden L (2015). Remarkable similarities of chromosomal rearrangements between primary human breast cancers and matched distant metastases as revealed by whole-genome sequencing. Oncotarget.

[35] Menghi F, Inaki K, Woo X, Kumar PA, Grzeda KR, Malhotra A, Yadav V, Kim H, Marquez EJ, Ucar D, Shreckengast PT, Wagner JP, MacIntyre G (2016). The tandem duplicator phenotype as a distinct genomic configuration in cancer. Proceedings of the National Academy of Sciences of the United States of America.

[36] Singh RR, Mehrotra M, Chen H, Almohammedsalim AA, Sahin A, Bousamra A, Patel K, Routbort MJ, Lu X, Abraham R, Mishra BM, Virani S, Medeiros L (2016). Comprehensive Screening of Gene Copy Number Aberrations in Formalin-fixed Paraffin-embedded Solid Tumors Using Molecular Inversion Probe-based SNP Array. Journal of Molecular Diagnostics.

[37] Koudelakova V, Trojanec R, Vrbkova J, Donevska S, Bouchalova K, Kolar Z, Varanasi L, Hajduch M (2016). Frequency of chromosome 17 polysomy in relation to CEP17 copy number in a large breast cancer cohort. Genes, chromosomes & cancer.

[38] Tse CH, Hwang HC, Goldstein LC, Kandalaft PL, Wiley JC, Kussick SJ, Gown AM (2011). Determining true HER2 gene status in breast cancers with polysomy by using alternative chromosome 17 reference genes: implications for anti-HER2 targeted therapy. Journal of clinical oncology.

[39] Marchio C, Lambros MB, Gugliotta P, Di Cantogno LV, Botta C, Pasini B, Tan DS, Mackay A, Fenwick K, Tamber N, Bussolati G, Ashworth A, Reis-Filho JS (2009). Does chromosome 17 centromere copy number predict polysomy in breast cancer? A fluorescence in situ hybridization and microarray-based CGH analysis. The Journal of pathology.

[40] Yeh IT, Martin MA, Robetorye RS, Bolla AR, McCaskill C, Shah RK, Gorre ME, Mohammed MS, Gunn SR (2009). Clinical validation of an array CGH test for HER2 status in breast cancer reveals that polysomy 17 is a rare event. Modern pathology.

[41] Frampton GM, Fichtenholtz A, Otto GA, Wang K, Downing SR, He J, Schnall-Levin M, White J, Sanford EM, An P, Sun J, Juhn F, Brennan K (2013). Development and validation of a clinical cancer genomic profiling test based on massively parallel DNA sequencing. Nature biotechnology.

[42] S Elbauomy Elsheikh, Green AR, Lambros MB, Turner NC, Grainge MJ, Powe D, Ellis IO, Reis-Filho JS (2007). FGFR1 amplification in breast carcinomas: a chromogenic in situ hybridisation analysis. Breast cancer research.

[43] Jacquemier J, Adelaide J, Parc P, Penault-Llorca F, Planche J, deLapeyriere O, Birnbaum D (1994). Expression of the FGFR1 gene in human breast-carcinoma cells. International journal of cancer.

[44] Helsten T, Elkin S, Arthur E, Tomson BN, Carter J, Kurzrock R (2016). The FGFR Landscape in Cancer: Analysis of 4,853 Tumors by Next-Generation Sequencing. Clinical cancer research.

[45] Edge SB, American Joint Committee on Cancer (2010). AJCC cancer staging manual.

[46] Singh RR, Patel KP, Routbort MJ, Reddy NG, Barkoh BA, Handal B, Kanagal-Shamanna R, Greaves WO, Medeiros LJ, Aldape KD, Luthra R (2013). Clinical validation of a next-generation sequencing screen for mutational hotspots in 46 cancer-related genes. The Journal of molecular diagnostics.

[47] Singh RR, Patel KP, Routbort MJ, Aldape K, Lu X, Manekia J, Abraham R, Reddy NG, Barkoh BA, Veliyathu J, Medeiros LJ, Luthra R (2014). Clinical massively parallel next-generation sequencing analysis of 409 cancer-related genes for mutations and copy number variations in solid tumours. Brit J Cancer.

[48] Wang L, Zehir A, Sadowska J, Zhou N, Rosenblum M, Busam K, Agaram N, Travis W, Arcila M, Dogan S, Berger MF, Cheng DT, Ladanyi M (2015). Consistent copy number changes and recurrent PRKAR1A mutations distinguish Melanotic Schwannomas from Melanomas: SNP-array and next generation sequencing analysis. Genes, chromosomes & cancer.

[49] Chin SF, Teschendorff AE, Marioni JC, Wang Y, Barbosa-Morais NL, Thorne NP, Costa JL, Pinder SE, van de Wiel MA, Green AR, Ellis IO, Porter PL, Tavare S (2007). High-resolution aCGH and expression profiling identifies a novel genomic subtype of ER negative breast cancer. Genome biology.

