# Supplementary material for: Genome-wide copy number aberrations and HER2 and FGFR1 alterations in primary breast cancer by molecular inversion probe microarray
Source: Oncotarget. 2017 Jan 24;8(7):10845–57. doi: 10.18632/oncotarget.14802 (PMC5355228; doi:10.18632/oncotarget.14802)
Supplement: Supplementary file 1 [file oncotarget-08-10845-s001.pdf]

Genome-wide copy number aberrations and *HER2* and *FGFR1* alterations in primary breast cancer by molecular inversion probe microarray

Supplementary Table 1: Comparison of NGS and MIP microarray in detecting amplification of *HER2* and *FGFR1* in breast cancer.

	NGS	MIP microarray		P value
		Amplified	Not amplified	
HER2	Amplified (n=3)	3	0	<0.01
	Not amplified (n=14)	1*	13	
FGFR1	Amplified (n=4)	4	0	<0.01
	Not amplified (n=13)	1*	12	

*The designated cut-off for gene amplification for tumor cells is 4 copies by MIP microarray and more than 7 copies by NGS due to high background noise in NGS tracing. One case with 4 copies of *FGFR1* and one separate case with 6 copies of *HER2* detected by MIP microarray could not be detected by NGS.

Abbreviations: *FGFR1*, fibroblast growth factor receptor 1; *HER2*, human epidermal growth factor receptor 2; MIP, molecular inversion probe; NGS, next generation sequencing.
